# Myocardial perfusion 3-Tesla cardiac magnetic resonance vs. exercise electrocardiogram for diagnosics of coronary artery disease

**DOI:** 10.1186/1532-429X-13-S1-P74

**Published:** 2011-02-02

**Authors:** Uwe Speiser, Achmed Abbas, Stefanie Jellinghaus, Burkhard Sievers, Ruth H Strasser, Steffen Schön

**Affiliations:** 1 Dresden University of Technology, Dresden, Germany

## Introduction

Myocardial perfusion cardiac magnetic resonance imaging (MRI) is established as a high sensitive procedure to diagnose coronary artery disease (CAD). In practice exercise electrocardigram (XECG) is widely used as non invasive method to detect relevant coronary stenosis (>50%).

The aim of this study was to investigate the diagnostic perfomance of adenosin stress 3-Tesla cardiac MRI for verification of ischaemia at coronary artery disease in comparison to XECG referred to coronary angiography as gold standard.

## Methods

We included patients who received XECG, stress cardiac MRI and accomplished invasive coronary diagnostics. The MRI measurements at 3.0 T based on cine-mode-sequences in short axes images, rest and adenosin stress (140 µg/kg bw/min) perfusion and late gadolinium enhancement imaging (LGE, Magnevist 0,2mmol/kg bw). Myocardial ischemia was defined as an area of perfusion deficit at stress MRI with negative late enhancement in areas of hypoperfusion (Panels A and B).

XECG was carried out on bicycle ergometer with a standardised protocol.

## Results

40 patients (63±11 years) were analysed prospectively, ten patients were assumed to have CAD, 30 had known CAD and were supposed to have a progress. A myocardial infarction in history was known at 14 patients,

XECG showed pathological findings in 24 cases (60%), twelve of these had angina or severe dyspnea, eleven patients had significant horizontal or down sloping ST-depression, one patient had an unsustained ventricular tachycardia.

Coronary angiography investigated a significant CAD (stenosis ≥50%) at 30 patients.

10 with normal XECG had relevant CAD. Sensitivity of XECG was 67%, specifity 60%. The positive predictive value (PPV) was calculated with 83%, the negative predictive value (NPV) was 37%.

Adenosin stress cardiac MRI revealed perfusion deficits in 32 patients (80%). The sensitivity of MRI concerning coronary stenosis ≥50% was 93%, the specifity 60%. Two patients with inconspicuous adenosin stress cardiac MRI had significant CAD. The PPV of MRI was 88%, the NPV 75%. All 14 patients with prior myocardial infarction were detected by LGE-sequence (both sensitivity and specifity 100%).

## Conclusion

Myocardial perfusion magnetic resonance imaging at 3.0 Tesla is superior to exercise electrocardigram to diagnose relevant (stenosis ≥50%) coronary artery disease. Cardiac MRI could also demarcate all patients with prior myocardial infarction by late enhancement.

